# Release of Ca^2+^ from the endoplasmic reticulum and its subsequent influx into mitochondria trigger celastrol-induced paraptosis in cancer cells

**DOI:** 10.18632/oncotarget.2256

**Published:** 2014-07-25

**Authors:** Mi Jin Yoon, A Reum Lee, Soo Ah Jeong, You-Sun Kim, Jin Yeop Kim, Yong-Jun Kwon, Kyeong Sook Choi

**Affiliations:** ^1^ Department of Biochemistry, Department of Biomedical Sciences, Ajou University School of Medicine, Suwon, Korea; ^2^ Discovery Biology Group, Institut Pasteur Korea, Sampyeong-dong 696, Bundang-gu, Seongnam-si, Gyeonggi-do, South Korea

**Keywords:** paraptosis, celastrol, endoplasmic reticulum, mitochondria, Ca^2+^

## Abstract

Celastrol, a triterpene extracted from the Chinese “Thunder of God Vine”, is known to have anticancer activity, but its underlying mechanism is not completely understood. In this study, we show that celastrol kills several breast and colon cancer cell lines by induction of paraptosis, a cell death mode characterized by extensive vacuolization that arises via dilation of the endoplasmic reticulum (ER) and mitochondria. Celastrol treatment markedly increased mitochondrial Ca^2+^ levels and induced ER stress via proteasome inhibition in these cells. Both MCU (mitochondrial Ca^2+^ uniporter) knockdown and pretreatment with ruthenium red, an inhibitor of MCU, inhibited celastrol-induced mitochondrial Ca^2+^ uptake, dilation of mitochondria/ER, accumulation of poly-ubiquitinated proteins, and cell death in MDA-MB 435S cells. Inhibition of the IP_3_ receptor (IP_3_R) with 2-aminoethoxydiphenyl borate (2-APB) also effectively blocked celastrol-induced mitochondrial Ca^2+^ accumulation and subsequent paraptotic events. Collectively, our results show that the IP_3_R-mediated release of Ca^2+^ from the ER and its subsequent MCU-mediated influx into mitochondria critically contribute to celastrol-induced paraptosis in cancer cells.

## INTRODUCTION

**Figure 1:**
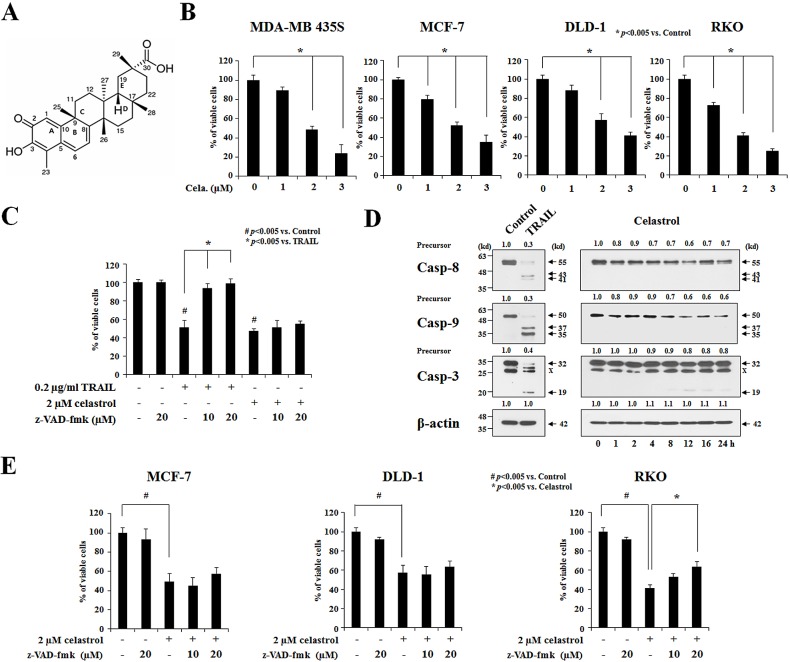
Apoptosis is not critically involved in the celastrol-induced cancer cell death **(A)** The chemical structure of celastrol. **(B)** Two breast cancer cell lines (MDA-MB 435S and MCF-7) and two colon cancer cell lines (DLD-1 and RKO) were treated with celastrol at the indicated concentrations for 24 h. Cellular viability was assessed using calcein-AM and EthD-1 to detect live and dead cells, respectively. **(C)** MDA-MB 435S cells were pretreated with the indicated concentrations of z-VAD-fmk for 30 min and further treated with 0.2 μg/ml TRAIL or 2 μM celastrol for 24 h. Cellular viability was assessed using calcein-AM and EthD-1. **(D)** MDA-MB 435S cells were treated with 0.2 μg/ml TRAIL for 24 h or 2 μM celastrol for the indicated time points. Whole cell extracts were prepared from the treated cells and subjected to Western blotting. β-actin was used as a loading control in Western blots. The fold change of protein levels compared to control (untreated cells) was determined by a densitometric analysis. **(E)** Cells were pretreated with the indicated concentrations of z-VAD-fmk for 30 min and further treated with or without 2 μM celastrol for 24 h. Cellular viability was assessed using calcein-AM and EthD-1.

Celastrol, a quinone methide triterpene, is a pharmacologically active compound derived from the Chinese medicinal plant, *Tripterygium wilfordii* [1]. Two carbons of celastrol, C_2_ of the A-ring and C_6_ of the B-ring (Figure 1A), reportedly show high susceptibilities for nucleophilic attack [2]. Celastrol can react with the nucleophilic thiol groups of cysteine residues and form covalent Michael adducts [3-6]. This seems to be the major mechanism through which celastrol can alter the functions of various proteins. Celastrol has traditionally been used to treat autoimmune diseases [7], chronic inflammation [8], asthma [9], and neurodegenerative diseases [10]. More recently, it has attracted interest as a potential anti-cancer agent, since it has been shown to inhibit proliferation and suppress the initiation, progression and metastasis of tumors in a wide variety of models *in vitro* and *in vivo* [11-14]. To date, the studies on the cancer-killing activity of celastrol have mainly focused on its ability to induce apoptosis [15,16]. In the present study, in contrast, we show that celastrol kills breast and colon cancer cell lines via inducing paraptosis. Despite recent improvements in anti-cancer therapies, inherent or acquired cellular resistance to various pro-apoptotic treatments often leads to therapeutic failure [17]. Thus, a better understanding of alternative, non-apoptotic cell death pathways, including paraptosis, may facilitate the design of novel therapeutics against malignant cancer cells that harbor defective apoptotic machineries. The term “paraptosis” was originally introduced to describe a form of programmed cell death that is morphologically and biochemically distinct from apoptosis [18,19]. It is characterized by: extensive cytoplasmic vacuolization that arises via swelling of the ER [19-21] and/or mitochondria [19,21,22]; the lack of characteristic apoptotic features, such as pyknosis, DNA fragmentation and caspase activation [19,21,23]; insensitivity to caspase inhibitors [18,24]; and overexpression of anti-apoptotic Bcl-2-like proteins [18,21,24]. Therefore, identification of agents that can induce paraptosis by targeting both mitochondria and the ER may provide a rational therapeutic strategy for effectively killing malignant cancer cells that resist apoptosis. However, the mechanisms underlying paraptosis, particularly the signals responsible for triggering dilation of mitochondria and the ER are still poorly defined. Observations that paraptosis can be inhibited by cycloheximide indicate that the paraptotic process requires protein synthesis [19,21,22,25]. MAP kinase activation has been associated with paraptosis induced by insulin-like growth factor I receptor (IGFIR) [18], curcumin [21,22], celastrol [25], and taxol [26], although the importance of the respective MAP kinase differs depending on the stimulus [18,21,22,25,26]. We recently showed that proteasomal dysfunction and the generation of mitochondrial superoxide are critical for the curcumin-induced dilation of mitochondria/ER and subsequent paraptotic cell death in breast cancer cells [21]. We propose here that the IP_3_R-mediated release of Ca^2+^ from the ER and its subsequent mitochondrial Ca^2+^ uniporter-mediated influx into mitochondria may critically contribute to extensive dilation of mitochondria and the ER, leading to celastrol-induced paraptotic cell death.

## RESULTS

### Neither apoptosis nor autophagy is critically involved in celastrol-induced cancer cell death

To investigate the anti-cancer effects of celastrol, we treated two breast cancer cell lines (MDA-MB 435S and MCF-7) and two colon cancer cell lines (DLD-1 and RKO) with various doses of celastrol for 24 h and performed cell viability assays using calcein-AM and EthD-1 to detect live and dead cells, respectively. We found that celastrol dose-dependently increased cell death over a range of 1-3 μM (Figure 1B). To test whether celastrol kills these cancer cells via apoptosis, we used the tumor necrosis factor-related apoptosis-inducing ligand (TRAIL), a cytokine that induces apoptosis by binding to the death receptors DR4 and DR5 [27] as a positive control. Treatment of MDA-MB 435S cells for 24 h with the apoptosis inducer TRAIL (0.2 μg/ml) effectively induced cell death (Figure 1C) in association with the effective proteolytic cleavage of caspase-8, -9, and -3 (Figure 1D). Pretreatment with z-VAD-fmk, a pan-caspase inhibitor, almost completely blocked TRAIL-induced cell death, but not celastrol-induced cell death in these cells (Figure 1C). Consistent with this, celastrol treatment was not accompanied by detectable proteolytic processing of cspase-8 or -9, except the minimal expression of p20 intermediate form of caspase-3 (Figure 1D). Furthermore, z-VAD-fmk pretreatment did not significantly inhibit celastrol-induced cell death in MCF-7 and DLD-1 cells, although it slightly attenuated cell death in RKO cells (Figure 1E). Moreover, chromatin condensation, DNA fragmentation, and PARP cleavage were frequently observed in MDA-MB 435S cells treated with TRAIL, as revealed by DAPI staining and immunostaining for cleaved PARP, but not in cells treated with celastrol (Supplementary Figure 1A). Immunocytochemical analysis further revealed that TRAIL induced the release of mitochondrial cytochrome *c*, whereas celastrol did not (Supplementary Figure 1B). Taken together, these results indicate that apoptosis may not critically contribute to the cytotoxicity of celastrol toward the tested cancer cell lines.

**Figure 2:**
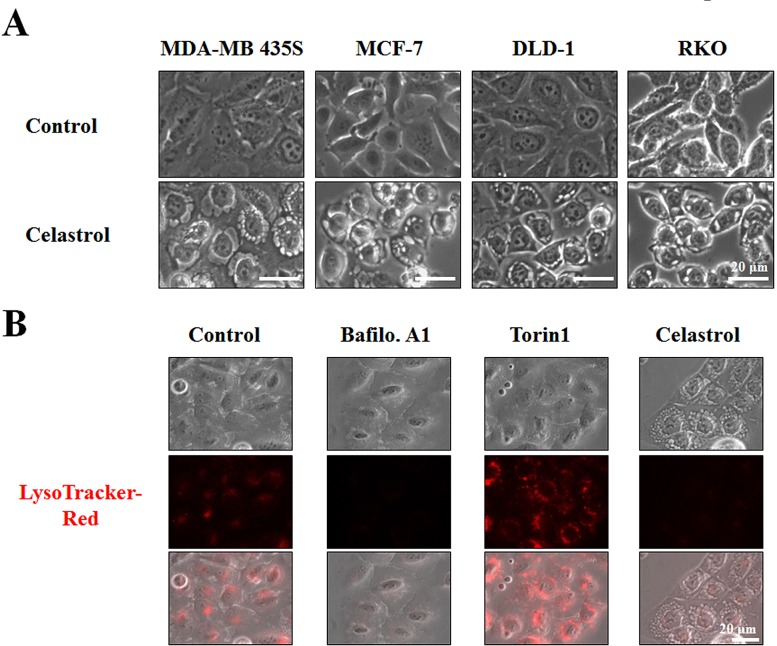
Celastrol induces vacuolation, but not lysosomal activation **(A)** Cells were treated with 2 μM celastrol for 8 h and observed under the phase contrast microscope. **(B)** MDA-MB 435S cells were left untreated or treated with 10 nM bafilomycin A1 (Baflo. A1), 1 μM Torin1, or 2 μM celastrol for 8 h, stained with 50 nM LysoTracker-Red for 20 min, and then observed under the phase contrast and a fluorescence microscope.

**Figure 3:**
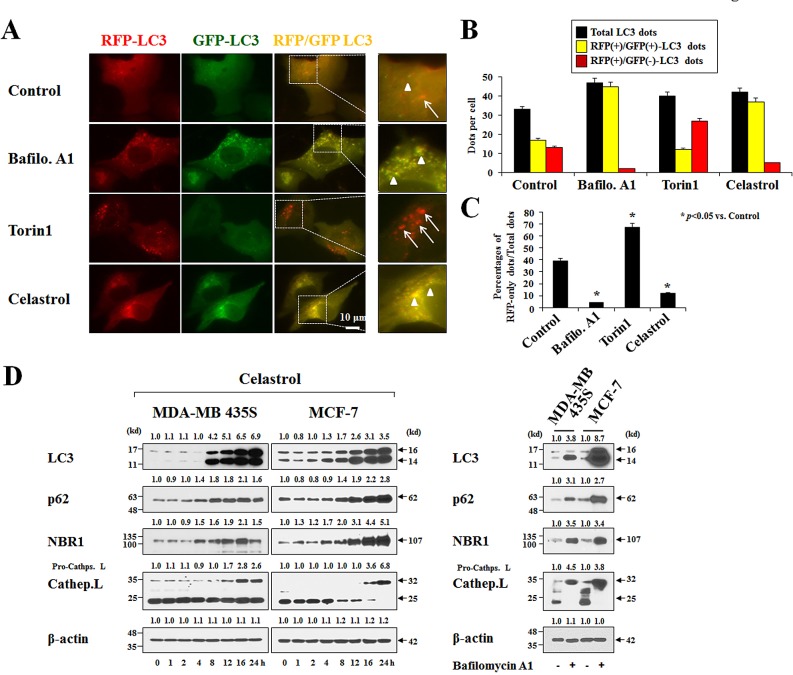
Celastrol inhibits autophagy **(A)** MDA-MB 435S cells transiently transfected with mRFP-GFP-LC3 plasmid for 24 h were further treated with 10 nM bafilomycin A1 (Baflo. A1), 1 μM Torin1, or 2 μM celastrol for 8 h. Representative fluorescence microscopic images are shown. Arrow heads: yellow dots (RFP(+)/GFP(+)-LC3 puncta), arrows: RFP-LC3-only dots (RFP(+)/GFP(-)-LC3 puncta). (B,C) Total, RFP(+)/GFP(+)-LC3, and RFP(+)/GFP(-)-LC3 dots were quantified and their percentages were calculated (>20 cells were counted in each experiment from at least three independent experiments. **(D)** Cells were treated with 2 μM celastrol for the indicated time points (*left*) or 10 nM bafilomycin A1 for 24 h (*right*). Whole cell extracts were prepared from the treated cells and subjected to Western blotting. β-actin was used as a loading control in Western blots. The relative expression levels were determined by the fold change of densitometric values in treated groups to the values in the control (untreated) group.

When we examined the cellular morphologies following celastrol treatment, we found that marked cellular vacuolation commonly preceded cell death in MDA-MB 435S, MCF-7, DLD-1 and RKO cells (Figure 2A). Therefore, we examined whether celastrol-induced vacuolation and subsequent cell death were associated with autophagy. First, we tested the possibility that celastrol induces lysosomal activation, a late step in autophagy, by staining with LysoTracker-Red. Treatment of MDA-MB 435S cells with Torin1, an autophagy inducer with mTOR (mechanistic target of rapamycin) inhibitory activity [28], markedly increased LysoTracker-Red staining, whereas bafilomycin A1, an autophagy inhibitor, substantially reduced it (Figure 2B). Interestingly, celastrol treatment, like bafilomycin A1 treatment, also inhibited LysoTracker-Red staining. We further measured autophagic flux activity in MDA-MB 435S cells employing the tandem fluorescent construct, mRFP/GFP-LC3 [29]. In this assay, RFP fluorescence is relatively stable in acidic compartments, whereas GFP fluorescence is rapidly quenched in such environments. Accordingly, mRFP/GFP-LC3 in mature autolysosomes will be detected as red puncta, whereas blocking autophagosome-lysosome fusion or suppressing lysosomal degradation (i.e., through an increase in lysosomal pH) will increase the number of yellow puncta [29]. We found that celastrol treatment increased the number of yellow puncta (RFP(+)/GFP(+)-LC3) similar to bafilomycin A1 treatment, whereas Torin1 treatment increased red puncta (RFP(+)/GFP(-)-LC3 puncta) (Figure 3A-3C). Time-course experiments showed that LC3 (both I and II form), as well as p62 [30] and NBR1 (Neighbor of Braca1 gene) [31], the substrates of autophagy, progressively accumulated in both MDA-MB 435S and MCF-7 cells treated with celastrol, similar to those obtained with bafilomycin A1 treatment (Figure 3D). Also similar to bafilomycin A1 treatment, celastrol inhibited the proteolytic processing of cathepsin L, a major lysosomal protease. These results would seem to suggest that celastrol might inhibit autophagy, possibly at the lysosomal step. However, we found that neither celastrol-induced cell death nor cellular vacuolation was affected not only by pretreatment with the autophagy inhibitors, 3-MA, bafilomycin A1, and chloroquine (CQ) but also by knockdown of ATG5, Beclin-1 and LAMP2 (Supplementary Figure 2A-2D). Furthermore, celastrol-induced cell death was not affected by the pretreatment with necrostatin-1, an inhibitor of necroptosis (Supplementary Figure 2E and 2F). Collectively, these results suggest that celastrol-induced vacuolation and subsequent cell death in MDA-MB 435S cells do not involve modulation of autophagy or necroptosis.

### Celastrol induces paraptosis in MDA-MB 435S cells

**Figure 4:**
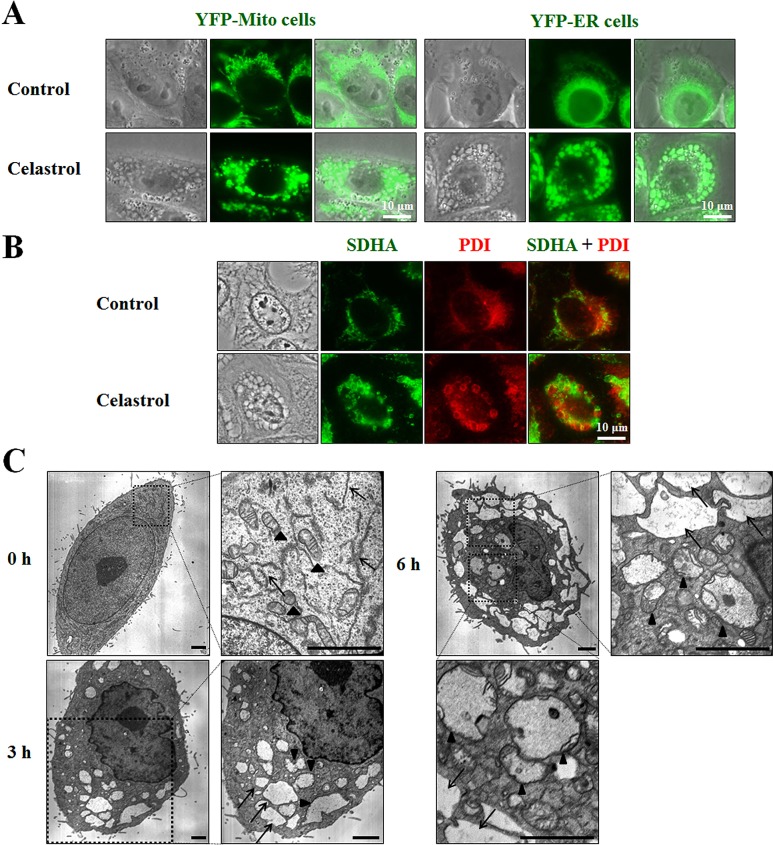
Celastrol-induced vacuoles are derived from mitochondria and the ER **(A)** YFP-Mito or YFP-ER cells treated with 2 μM celastrol for 3 h were observed under the phase contrast and fluorescence microscope. **(B)** MDA-MB 435S cells were treated with or without 2 μM celastrol for 3 h. Immunocytochemistry using anti-SDHA (green) and anti-PDI (red) antibodies was performed and the representative phase contrast and fluorescence microscopic images of cells are shown. **(C)** MDA-MB 435S cells were treated with 2 μM celastrol for the indicated time points and observed by transmission electron microscopy. Arrowheads indicate mitochondria and arrows denote the ER. Swelling and fusion of mitochondria and the ER are seen after celastrol treatment. Bars, 2 μm.

**Figure 5:**
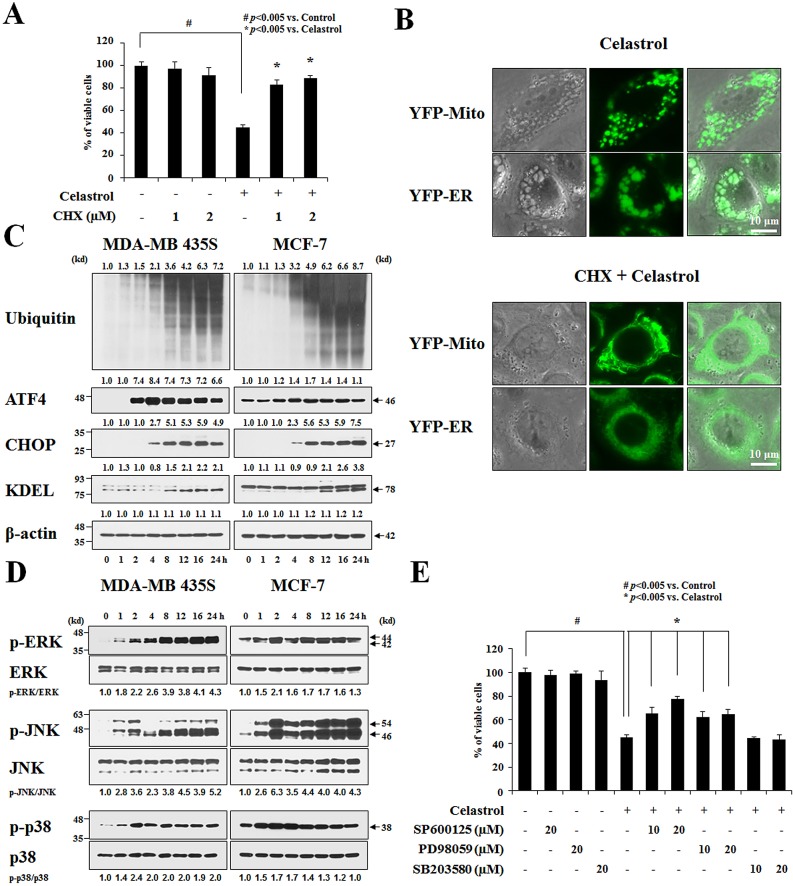
Celastrol induces paraptosis **(A)** MDA-MB 435S cells were left untreated or were pretreated with cycloheximide (CHX) at the indicated concentrations for 30 min and then treated with 2 μM celastrol for an additional 24 h in the continued presence of CHX. Cellular viability was assessed using calcein-AM and EthD-1. **(B)** YFP-Mito or YFP-ER cells were left untreated or were pretreated with 1 μM CHX and further treated with 2 μM celastrol for an additional 3 h in the continued presence of CHX. Cells were observed under the phase contrast and fluorescence microscope. **(C)** Cells were treated with 2 μM celastrol for the indicated time points and Western blotting was performed. β-actin was used as a loading control in Western blots. The relative expression levels were determined by the fold changes of densitometric values in treated groups to the values in the control (untreated) group. **(D)** Cells were treated with 2 μM celastrol for the indicated time points and Western blotting was performed. The relative phosphorylation levels of the respective MAP kinase were determined by the fold changes of densitometric values in treated groups to the values in the control group. Densitometric values for the phospho-proteins of interest were normalized for protein loading with their total proteins. Similar results were obtained from three independent experiments. **(E)** MDA-MB 435S cells were untreated or pretreated with the indicated specific inhibitors (SP600125, PD98059, and SB203580) at the indicated concentrations for 30 min and further treated with 2 μM celastrol for 24 h. Cellular viability was assessed using calcein-AM and EthD-1.

Next, to test whether the celastrol-induced vacuoles might originate from mitochondria and/or the ER, we used MDA-MB 435S sublines stably expressing fluorescence selectively in mitochondria or the ER, YFP-Mito cells and YFP-ER cells [21]. As shown in Figure 4A, mitochondria in untreated YFP-Mito cells exhibited a filamentous, elongated morphology, whereas the ER in untreated YFP-ER cells formed a reticulate structure. At 3 h after treatment with 2 μM celastrol, numerous fluorescent vacuoles were observed in both YFP-Mito and YFP-ER cells. Immunocytochemistry using specific antibodies against the subunit A of succinate dehydrogenase (SDHA), a mitochondrial protein, and protein disulfide-isomerase (PDI), an ER-resident protein, showed many small rings of SDHA expression and larger rings of PDI expression in MDA-MB 435S cells treated with celastrol for 3 h (Figure 4B). A more detailed electron microscopy revealed swollen mitochondria and the ER structures in MDA-MB 435S cells treated with 2 μM celastrol for 3 h. At 6 h of celastrol treatment, fusion among swollen mitochondria and the swollen ER had further progressed (Figure 4C). In contrast, untreated MDA-MB 435S cells possessed mitochondria with intact cristae and the ER structures with a reticular morphology. Collectively, these results indicate that celastrol induces paraptotic morphologies. We next sought biochemical evidence for the induction of paraptosis by celastrol. We first tested the effect of the protein synthesis inhibitor, cycloheximide, on celastrol-induced cell death. Pretreatment with cycloheximide (CHX) very effectively blocked cell death in celastrol-treated MDA-MB 435S cells (Figure 5A), and prevented mitochondrial/ER dilation in celastrol-treated YFP-Mito and YFP-ER cells (Figure 5B). Recently, we showed that proteasome dysfunction is required for dilation of mitochondria/ER and consequent paraptotic cell death [21,22]. Thus, we asked whether celastrol also impairs proteasome activity. We found that celastrol-treated cells progressively accumulated poly-ubiquitinated proteins (Figure 5C). Furthermore, celastrol treatment markedly increased the protein levels of ATF4, CHOP, and KDEL in MDA-MB 435S and MCF-7 cells, indicating that celastrol induces ER stress. When we examined the activity changes in MAP kinases, we found that ERKs were progressively activated in MDA-MB 435S cells treated with celastrol. In contrast, the activities of ERKs increased with a peak at 2 h of celastrol treatment and then slowly reduced in MCF-7 cells (Figure 5D). In both MDA-MB 435S and MCF-7 cells, JNKs demonstrated biphasic activation patterns following celastrol treatment, whereas p38 showed transient activation with a peak at 2 h after celastrol treatment. When we investigated the importance of these signals, we found that celastrol-induced cell death was significantly inhibited by SP600125-induced inhibition of JNK, inhibited by PD98059-induced inhibition of ERK at lesser extent, and unaffected by SB203580-induced inhibition of p38 (Figure 5E). Taken together, our results indicate that celastrol may induce paraptosis in the tested breast cancer cell lines.

### IP_3_R-mediated Ca^2+^ release and uniporter-mediated mitochondrial influx of Ca^2+^ are critical for celastrol-induced paraptosis

**Figure 6:**
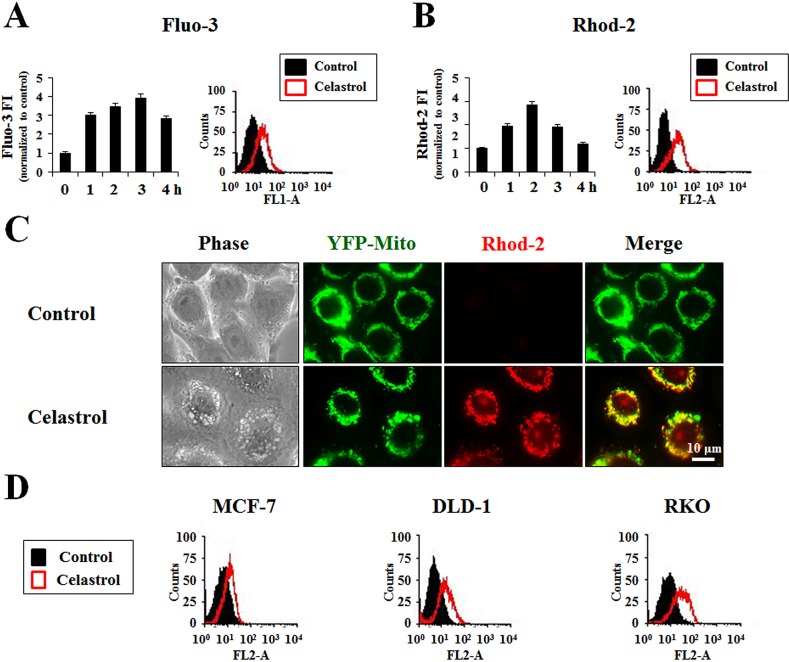
Celastrol induces mitochondrial Ca^2+^ uptake **(A)** MDA-MB 435S cells treated with 2 μM celastrol for the indicated time points were stained with 2.5 μM Fluo-3 and processed for FACS analysis. Fluo-3 fluorescence intensities (FI) in cells treated with 2 μM celastrol were compared with that of untreated cells and denoted in the graph (*left*). Histogram for the cells treated with 2 μM celastrol for 3 h is shown (*right*). X axis, fluorescence intensity, Y axis, relative number of cells. **(B)** MDA-MB 435S cells treated with or without 2 μM celastrol for the indicated time points were stained with 2.5 μM Rhod-2 and processed for FACS analysis. Rhod-2 fluorescence intensities (FI) were compared with that of untreated cells and denoted in the graph (*left*). Histogram for the cells treated with 2 μM celastrol for 2 h is shown (*right*). **(C)** YFP-Mito cells treated with or without 2 μM celastrol for 2 h were stained with 2.5 μM Rhod-2 and then observed under the phase contrast and fluorescence microscopy. **(D)** MCF-7, DLD-1 and RKO cells treated with 2 μM celastrol for 4 h. Treated cells were stained with 2.5 μM Rhod-2 and processed for FACS analysis. The representative histograms are shown. X axis, fluorescence intensity, Y axis, relative number of cells.

Since mitochondria and the ER are major reservoirs of intracellular Ca^2+^, we next tested whether their dilation following celastrol treatment was associated with disruptions in intracellular Ca^2+^ homeostasis. Flow cytometry using Fluo-3 (a cell-permeable Ca^2+^-indicator dye) demonstrated that treatment of MDA-MB 435S cells with celastrol dramatically increased the intracellular Ca^2+^ levels ([Ca^2+^]_i_), which peaked at 3 h post-treatment (Figure 6A). Furthermore, flow cytometry using Rhod-2 (an indicator dye for mitochondrial Ca^2+^) showed that celastrol treatment also increased the mitochondrial Ca^2+^ levels ([Ca^2+^]_m_), which peaked at 2 h post-treatment (Figure 6B). Fluorescence microscopy further confirmed the celastrol-induced increase in Rhod-2 staining intensity in mitochondria of YFP-Mito cells (Figure 6C). Moreover, flow cytometry using Rhod-2 showed that the celastrol-induced increase in [Ca^2+^]_m_ is common to MCF-7, DLD-1, and RKO cells (Figure 6D).

**Figure 7:**
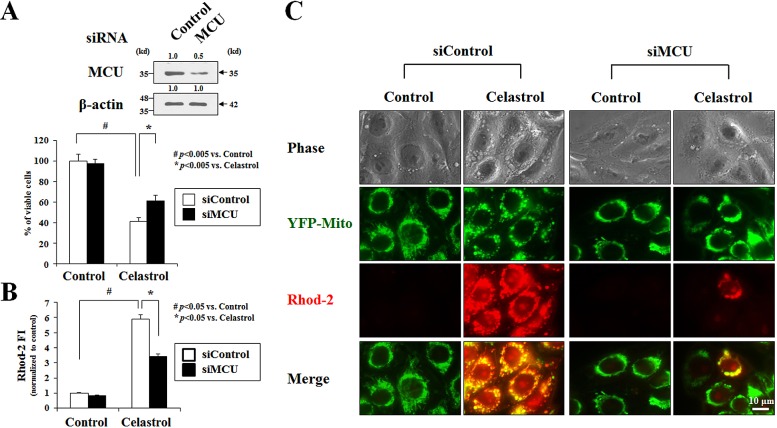
MCU knockdown inhibits celastrol-induced paraptosis **(A)** MDA-MB 435S cells were transfected with MCU siRNA and further treated with or without 2 μM celastrol for 24 h. Knockdown of MCU was confirmed by Western blotting using anti-MCU antibody. β-actin was used as a loading control in Western blots (*upper panel*). Cellular viability was assessed using calcein-AM and EthD-1 (*lower panel*). **(B)** MDA-MB 435S cells were transfected with MCU siRNA and further treated with or without 2 μM celastrol for 2 h. Cells were stained with 2.5 μM Rhod-2 and processed for FACS analysis. The fold changes of Rhod-2 fluorescence intensities (FI) are shown in the graph. **(C)** YFP-Mito cells were transfected with MCU siRNA and further treated with or without 2 μM celastrol for 2 h. Treated cells were stained with Rhod-2 and processed for the phase contrast and fluorescence microscopy.

**Figure 8:**
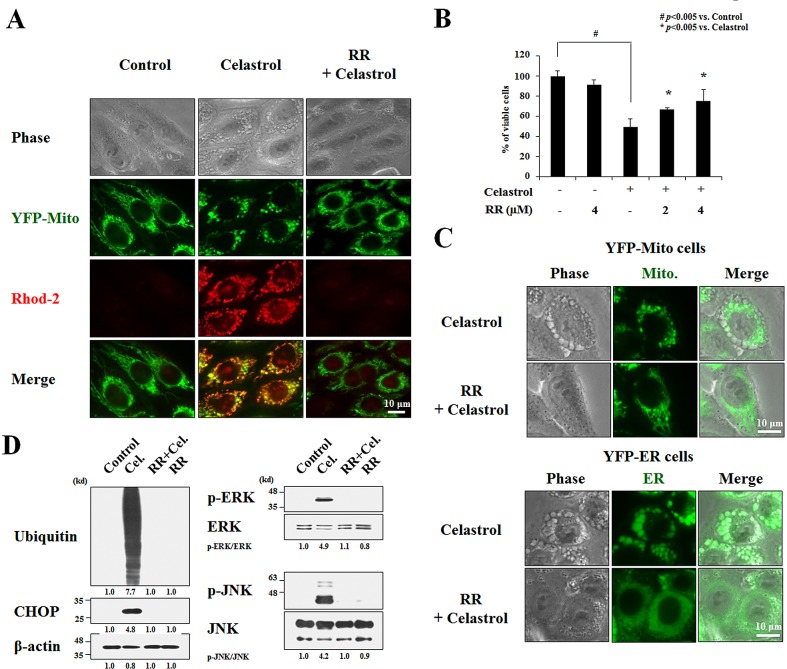
Inhibition of MCU blocks celastrol-induced paraptosis **(A)** YFP-Mito cells were pretreated with 4 μM ruthenium red (RR) and further treated with 2 μM celastrol for 2 h. Cells were stained with Rhod-2 and processed for the phase contrast and fluorescence microscopy. **(B)** MDA-MB 435S cells were pretreated with the indicated concentrations of ruthenium red and further treated with or without 2 μM celastrol for 24 h. Cellular viability was measured using calcein-AM and EthD-1. **(C)** YFP-Mito and YFP-ER cells were pretreated with 4 μM ruthenium red (RR), further treated with 2 μM celastrol for 3 h, and observed under the phase contrast and fluorescence microscope. **(D)** MDA-MB 435S cells were pretreated with 4 μM RR and further treated with 2 μM celastrol for 24 h followed by Western blotting. β-actin was used as a loading control in Western blots. The relative phosphorylation levels of the respective MAP kinase were determined by the fold changes of densitometric values in treated groups to the values in the control group. Densitometric values for the phospho-proteins of interest were normalized for protein loading with their total proteins. The relative expression levels of CHOP and ubiquitin were determined using densitometric analysis compared to untreated control.

Ca^2+^ reportedly enters mitochondria via the MCU when [Ca^2+^]_i_ are high [32]. Thus, we next tested whether inhibition of MCU affected celastrol-induced paraptosis. The functional role of the MCU in celastrol-induced cell death was investigated by knocking it down using small interfering RNA. We found that the cell death in YFP-Mito cells induced by 2 μM celastrol was significantly attenuated by transfection with MCU siRNA, despite the incomplete knockdown of MCU (Figure 7A). In addition, FACS analysis and fluorescence microscopy using Rhod-2 in these cells showed that mitochondrial Ca^2+^ accumulation and cellular vacuolation induced by treatment with 2 μM celastrol for 2 h were also markedly reduced by MCU knockdown (Figures 7B and 7C). Pretreatment with ruthenium red (RR), an inhibitor of uniporter-mediated mitochondrial Ca^2+^ uptake [33,34], also effectively blocked the celastrol-induced increase in [Ca^2+^]_m_ in YFP-Mito cells (Figure 8A) and celastrol-induced death of MDA-MB 435S cells (Figure 8B). RR pretreatment also inhibited the dilation of mitochondria and the ER in YFP-Mito and YFP-ER cells (Figure 8C). Furthermore, RR pretreatment markedly inhibited celastrol-induced the accumulations of poly-ubiquitinated proteins, CHOP, activated ERK, and activated JNK (Figure 8D). We further tested whether treatment with kaempferol, an activator of the MCU [35], could sensitize MDA-MB 435S cells treated with the low-dose celastrol to paraptotic cell death. Compared to treatment with subtoxic dose (20 μM) of kaempferol or low-dose (1 μM) celastrol alone, combined treatment with kaempferol and celastrol for 4 h markedly increased [Ca^2+^]_m_ and cellular vacuolation in YFP-Mito cells (Supplementary Figure 3A). In addition, co-treatment with kaempferol dose-dependently enhanced the death of MDA-MB 435S cells treated with 1 μM celastrol, compared with cells treated with 1 μM celastrol alone (Supplementary Figure 3B). Collectively, these observations indicate that MCU-mediated mitochondrial Ca^2+^ uptake may play a critical role in celastrol-induced paraptosis.

**Figure 9:**
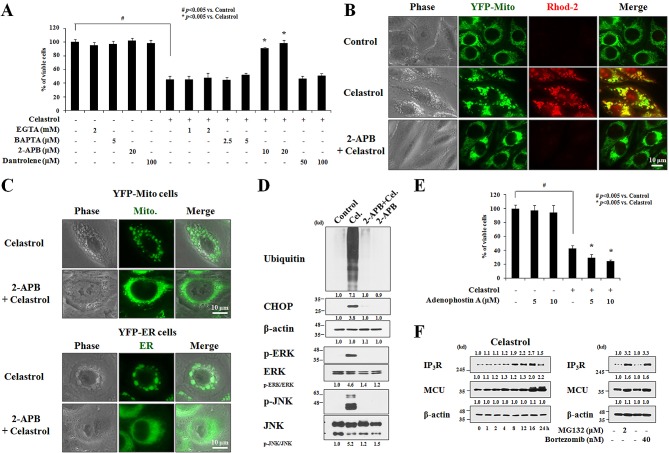
IP^3^R-mediated Ca^2+^ release from the ER is critical for celastrol-induced paraptosis **(A)** MDA-MB 435S cells were pretreated with the indicated concentrations of extracellular Ca^2+^ chelators (EGTA and BAPTA), 2-APB, and dantrolene for 30 min and further treated with or without 2 μM celastrol for 24 h. Cellular viability was measured using calcein-AM and EthD-1. **(B)** YFP-Mito cells were pretreated with 20 μM 2-APB and further treated with 2 μM celastrol for 2 h. Cells were stained with Rhod-2 and processed for the phase contrast and fluorescence microscopy. **(C)** YFP-Mito and YFP-ER cells were pretreated with 20 μM 2-APB, further treated with 2 μM celastrol for 3 h, and observed under the phase contrast and fluorescence microscope. **(D)** MDA-MB 435S cells were pretreated with 20 μM 2-APB and further treated with 2 μM celastrol for 24 h followed by Western blotting. β-actin was used as a loading control in Western blots. The relative phosphorylation levels of the respective MAP kinase were determined by the fold changes of densitometric values in treated groups to the values in the control group. Densitometric values for the phospho-proteins of interest were normalized for protein loading with their total proteins. The relative expression levels of CHOP and ubiquitin were determined using densitometric analysis compared to untreated control. **(E)** MDA-MB 435S cells were pretreated with the indicated concentrations of adenophostin A and further treated with or without 2 μM celastrol for 24 h. Cellular viability was measured using calcein-AM and EthD-1. **(F)** MDA-MB 435S cells were treated with 2 μM celastrol for the indicated time points (*left*), 2 μM MG132 or 40 nM bortezomib for 24 h (*right*) and Western blotting of IP_3_R, MCU, and β-actin was performed. Compared to control (untreated cells), the fold change of protein levels was determined by a densitometric analysis.

**Figure 10:**
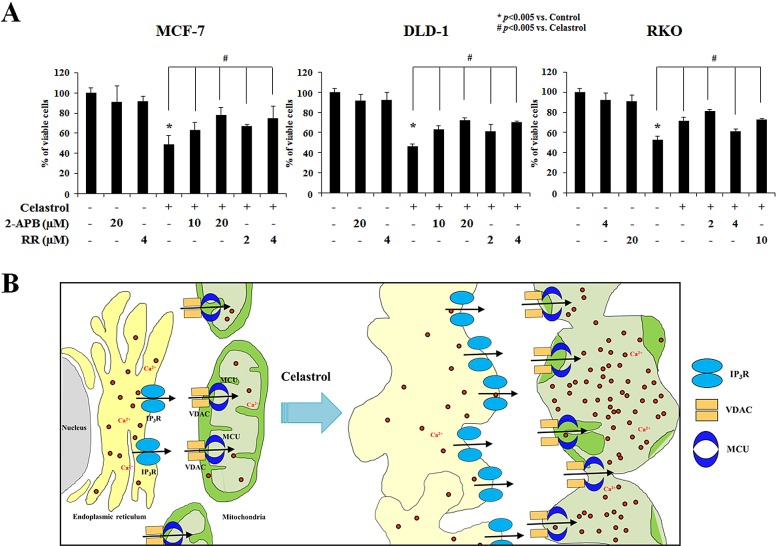
Celastrol induces paraptosis via IP3R-mediated Ca^2+^ release from the ER and MCU-mediated mitochondrial Ca^2+^ influx in cancer cells **(A)** Cells were pretreated with the indicated concentrations of RR or 2-APB for 30 min and further treated with 2 μM celastrol for 24 h. Cellular viability was assessed using calcein-AM and EthD-1. **(B)** Hypothetical scheme of celastrol-induced paraptosis. Celastrol triggers IP_3_R-mediated Ca^2+^ release from the ER and subsequently MCU-mediated Ca^2+^ influx into mitochondria, leading to the dilations of mitochondria/ER and cell death.

Next, we assessed the contribution of extracellular Ca^2+^ and intracellular Ca^2+^ stores to celastrol-induced mitochondrial Ca^2+^ accumulation. Since pretreatment with extracellular Ca^2+^ chelators, including EGTA or BAPTA, did not appear to alter celastrol-induced cell death (Figure 9A), we tested whether Ca^2+^ release from the ER contributed to celastrol-induced paraptosis. Experiments using specific inhibitors of two major Ca^2+^ release receptors in the ER, the IP_3_ receptor (IP_3_R) and the ryanodine receptor (RyR) [36], showed that celastrol-induced cell death was very effectively inhibited by 2-APB, a specific inhibitor of IP_3_R [37], but not by dantrolene, a specific inhibitor of the RyR [38] (Figure 9A). Staining of YFP-Mito cells with Rhod-2 also showed that 2-APB markedly reduced the celastrol-induced increase in [Ca^2+^]_m_ (Figure 9B). Furthermore, 2-APB effectively inhibits celastrol-induced dilation of mitochondria and the ER in YFP-Mito and YFP-ER cells, respectively (Figure 9C). These results suggest that pretreatment with 2-APB effectively inhibits the celastrol-induced cell death by inhibiting the IP_3_R-mediated release of Ca^2+^ from the ER and the subsequent MCU-mediated influx of Ca^2+^ into mitochondria. In addition, 2-APB pretreatment markedly inhibited celastrol-induced the accumulations of poly-ubiquitinated proteins, CHOP, activated ERK, and activated JNK (Figure 9D). The importance of IP_3_R-mediated Ca^2+^ release from the ER in celastrol-induced paraptosis was further tested using adenophostin A, an agonist of IP_3_R [39]. We found that co-treatment of MDA-MB 435S cells with adenophostin A dose-dependently enhanced cell death, when combined with 2 μM celastrol (Figure 9E). Fluorescence microscopy using Rhod-2 showed that co-treatment with 10 μM adenophostin A accelerated and enhanced celastrol-induced increase in [Ca^2+^]_m_ in cells treated with 2 μM celastrol (Supplementary Figure 4). Notably, the levels of IP_3_R and MCU protein increased following celastrol treatment (Figure 9F) and similar to celastrol, MG132 or bortezomib also increased IP_3_R and MCU protein levels in MDA-MB 435S cells (Figure 9F). These results suggest that the upregulation of IP_3_R and MCU as a consequence of celastrol-induced proteasome inhibition may contribute to the release of Ca^2+^ from the ER, Ca^2+^ influx into mitochondria, and subsequent paraptotic events. Finally, we found that pretreatment with RR or 2-APB significantly and dose-dependently inhibited celastrol-induced cell death also in MCF-7, DLD-1, and RKO cells (Figure 10A). In sum, we herein show for the first time that celastrol-induced paraptotic cell death in cancer cells is triggered by IP_3_R-mediated Ca^2+^ release from the ER and uniporter-mediated mitochondrial Ca^2+^ influx which collectively trigger dilation of mitochondria and the ER (Figure 10B).

## DISCUSSION

Accumulating evidence suggests that although the elimination of malignant cancer cells often depends on classical apoptotic pathways (mitochondrial and/or death-receptor pathways), alternative apoptotic and non-apoptotic pathways may effectively contribute to tumor cell death. In addition, researchers have speculated that the relative sensitivity of mitochondria/ER in cancer cells to oxidative stress and ER stress, compared to normal cells, could be exploited for the rational design of cancer therapeutics [40-43]. Thus, induction of paraptosis, the cell death mode that targets both mitochondria and the ER, may offer an attractive way to effectively kill malignant cancer cells that are resistant to conventional pro-apoptotic cancer therapies. In the present study, we show that celastrol effectively kills MDA-MB 435S, MCF-7, DLD-1, and RKO cancer cells via induction of paraptosis. During celastrol-induced cell death, autophagy was inhibited at lysosomal step and apoptosis was minimally involved. Similar to our results, Boridy S *et al*. showed that combination with autophagy inhibitor did not sensitize cells to celastrol-mediated cytotoxicity and celastrol-induced paraptosis in glioma cells occurred independently of apoptosis [44]. In contrast, celastrol was reported to induce paraptosis, autophagy, and apoptosis in HeLa cells [25], suggesting the possibility that celastrol may induce different cellular fates other than paraptosis, depending on cell types or cellular context. Celastrol treatment was found to induce extensive swelling and fusion of mitochondria and the ER, eventually leading to the formation of a few megamitochondria and substantially expanded ER structures. Based on these findings, we sought to decipher the key signals responsible for the dilation of mitochondria and the ER during celastrol-induced paraptosis. Here, we found that celastrol induced marked accumulation of poly-ubiquitinated proteins and various ER-stress marker proteins, including ATF4, CHOP and KDEL. Cycloheximide pretreatment almost completely blocked celastrol-induced vacuolation and subsequent cell death, suggesting that protein synthesis is required for the treatment-induced dilation of mitochondria and the ER.

The ER and mitochondria interact both physiologically and functionally, at least in part via Ca^2+^ signaling [45]. Mitochondria are juxtaposed to the ER in microdomains called mitochondria-associated ER membranes (MAMs) [46]. This physical association enables highly efficient inter-organelle communication, allowing mitochondria to tightly couple and coordinate Ca^2+^ fluxes with the ER and act as cellular sentinels for the ER-mediated Ca^2+^ signals [47]. The loss of Ca^2+^ homeostasis (e.g., cellular Ca^2+^ overload) or changes in Ca^2+^ distribution within intracellular compartments can lead to cell death [48]. Thus, we examined whether Ca^2+^ was involved in celastrol-induced mitochondria/ER dilation and subsequent cell death. We found that celastrol markedly increased [Ca^2+^]_i_, particularly [Ca^2+^]_m_, in our tested cancer cells. While the physiological [Ca^2+^]_m_ signal regulates [Ca^2+^]_c_ and stimulates oxidative metabolism, excess mitochondrial Ca^2+^ accumulation causes cell stress to leading to cell death [49]. The MCU is a major Ca^2+^ entry pathway through the mitochondrial inner membrane [50]. In our study, not only MCU knockdown but also RR pretreatment inhibited celastrol-induced mitochondrial Ca^2+^ accumulation, dilation of mitochondria and the ER, and cell death. In addition, kaempferol, a MCU activator, sensitized breast cancer cells treated with the low-dose celastrol to paraptosis. Taken together, these results suggest that mitochondrial Ca^2+^ uptake via MCU plays a critical role in celastrol-induced paraptosis.

An investigation of the source of Ca^2+^ responsible for mitochondrial Ca^2+^ overload following celastrol treatment showed that two extracellular Ca^2+^ chelators, BAPTA and EGTA, failed to block celastrol-induced cell death, suggesting the involvement of intracellular stores. Under normal physiological conditions, the bulk of Ca^2+^ resides within the ER lumen. The most important molecular component of the Ca^2+^-handling machinery in the ER is the IP_3_R, which mediates the release of Ca^2+^ following binding of its ligand IP_3_ [36]. IP_3_Rs are primarily clustered in MAM regions, which function as the primary subcellular microdomains of Ca^2+^ transfer from the ER to mitochondria [46]. In the present study, following results support the importance of the IP_3_R in celastrol-induced paraptosis: a) pretreatment of MDA-MB 435S cells with 2-APB, an IP_3_R antagonist, inhibited celastrol-induced mitochondrial Ca^2+^ accumulation, mitochondrial and the ER dilation, and cell death; b) co-treatment with adenophostin A potentiated celastrol-induced mitochondrial Ca^2+^ accumulation and cell death; c) celastrol treatment increased IP_3_R protein levels.

Although 2-APB was initially characterized as an antagonist of IP_3_Rs, increasing evidence has shown that 2-APB may be a powerful modifier of store-operated Ca^2+^ entry (SOCE) channels [51]. Low levels of 2-APB (1-10 μM) were shown to rapidly and profoundly stimulate Ca^2+^ entry, whereas higher levels of 2-APB (25-75 μM) induced a transient increase followed by complete inhibition [52]. Therefore, we presume that SOCE may not be critically involved in celastrol-induced paraptosis, since 10-20 μM 2-APB very effectively blocked the celastrol-induced increase in intracellular Ca^2+^ levels and cell death. IP_3_Rs are known to be polyubiquitinated, and it has been shown that proteasome inhibitors block their down-regulation [53]. In our study, celastrol increased the protein levels of not only IP_3_R but also MCU, similar to MG132 and bortezomib. Therefore, we speculate that upregulation of both IP_3_R and MCU as a result of celastrol-induced proteasome inhibition may contribute to the enhanced release of Ca^2+^ from the ER and subsequent mitochondrial Ca^2+^ influx.

The release of Ca^2+^ from the ER to the mitochondria is considered to be the major event leading to mitochondria swelling [54]. Our own unpublished data indicate that treatment of breast cancer cells with thapsigargin (an agent that reduces Ca^2+^ levels in the ER) induces transient swelling of mitochondria, but not the ER (data not shown). Giant ER-derived vacuoles have been increasingly recognized as indicating perturbation of the functional link between the ER and the proteasome [22,55,56]. Enhanced accumulation of misfolded proteins in cancer cells increases the dependence of cells on optimal proteasomal function [57], and failure of the proteasomal machinery leads to further accumulation of misfolded proteins in the ER and cytoplasm [58]. Although the mechanism underlying ER vacuolization has not been extensively investigated, Mimnaugh et al. [55] proposed that accumulation of misfolded proteins trapped within the ER could exert an osmotic force, inducing an influx of water from the cytoplasm and distending the ER luminal space into vacuoles. Celastrol has the potential to covalently modify sulfhydryl groups, causing protein misfolding [6], as well as the ability to inhibit proteasome activity [2]. Therefore, a massive buildup of misfolded proteins within the ER lumen may critically contribute to the ER vacuolization observed following celastrol treatment. This hypothesis is supported by our finding that the protein synthesis inhibitor, cycloheximide, protects breast cancer cells from celastrol-induced dilation of the ER and mitochondria and subsequent cell death. The ER controls the synthesis, folding and processing of proteins. Since a high concentration of Ca^2+^ in the ER is essential to the function of chaperones [e.g., glucose-regulated protein (GRP) 78 [59] and the processing of luminal proteins [60], depletion of ER Ca^2+^ levels will trigger the accumulation of misfolded proteins within the ER by impairing chaperone activity [59,61] and protein processing [60]. Therefore, we speculate that the celastrol-induced release of ER Ca^2+^ via IP_3_R contributes to the further accumulation of misfolded proteins in the ER and expansion of ER-derived vacuoles. This is supported by our observation that 2-APB pretreatment inhibited the celastrol-induced accumulation of poly-ubiquitinated proteins, dilation of mitochondria and the ER, and subsequent cell death. In addition, we cannot exclude the possibility that mitochondrial uptake of Ca^2+^ by celastrol, probably indirectly, may inhibit the proteasomal activity and/or ER structure, since RR pretreatment also inhibited the celastrol-induced accumulation of poly-ubiquitinated proteins and dilation of the ER, besides the dilation of mitochondria.

In sum, our results show that mitochondria and the ER cooperate to signal celastrol-induced paraptosis via Ca^2+^.

## MATERIALS AND METHODS

### Chemicals and antibodies

Celastrol, cycloheximide (CHX), 3-methyladenine (3-MA), bafilomycin A1, chloroquine (CQ), ethylene glycol tetraacetic acid (EGTA), 1,2-bis(o-aminophenoxy) ethane-N,N,N'N'-tetraacetic acid (BAPTA), 1,2-bis(o-aminophenoxy)ethane-N,N,N'N'-tetraacetic acid acetoxymethyl ester (BAPTA-AM), ruthenium red, and MG132 were purchased from Sigma-Aldrich. Torin1 was purchased from Selleckchem. LysoTracker-Red, Rhod-2-AM, Fluo-3-AM, calcein- acetoxymethyl ester (calcein-AM), and ethidium homodimer-1 (EthD-1) were from Molecular Probes. 2-aminoethosxydiphenyl borate (2-APB), PD98059, SB203580, and SP600125 were obtained from Calbiochem. Dantrolene was obtained from Alexis Biochemicals. The following antibodies were used: anti-β-actin (Abcam); anti-ubiquitin, ATF4, and IP_3_R (Santa Cruz Biotechnologies); anti-caspase-8, caspase-3, and KDEL (Stressgen); anti-caspase-9 (Novus Biologicals); anti-p62 and anti-cathepsin L (BD Biosciences); anti-MCU (Sigma-Aldrich); anti-NBR1 (Abnova); anti-LC3B, CHOP, phospho-ERK1/2, total ERK1/2, phospho-JNK, total JNK, phospho-p38, and total p38 (Cell Signaling); HRP-conjugated anti-rabbit IgG and HRP-conjugated anti-mouse IgG (Molecular Probes).

### Cell Culture

The MDA-MB 435S and MCF-7 human breast cancer cells and DLD-1 and RKO colon cancer cells were purchased from American Type Culture Collection. Cells were cultured in DMEM supplemented with 10% fetal bovine serum (FBS) and antibiotics (GIBCO-BRL) and incubated in 5% CO_2_ at 37°C.

### Measurement of cellular viability

Cell viability was assessed by double labeling of cells with 2 μM calcein-AM and 4 μM EthD-1. The calcein-positive live cells and EthD-1-positive dead cells were visualized using a fluorescence microscope (Axiovert 200M; Carl Zeiss) equipped with Zeiss filter set #46 (excitation band pass, 500/20 nm; emission band pass, 535/30 nm) and #20 (excitation band pass, 546/12 nm; emission band pass, 575-640 nm) and counted.

### Western blotting

Western blotting was performed as described in our previous studies [10]. The representative results from at least three independent experiments are shown. The respective protein band intensity was quantified by densitometric analysis using the NIH ImageJ program. The relative expression levels were determined by the fold change of densitometric values in treated groups to the values in the control group.

### Immunocytochemistry

After treatments, cells were fixed with acetone/methanol (1:1) for 5 min at -20°C and blocking in 5% BSA in PBS for 30 min. Fixed cells were incubated overnight at 4°C with primary antibody [anti-SDHA (1:500, mouse, Invitrogen) and anti-PDI (1:500, rabbit, Stressgen) diluted in PBS and then washed three times in PBS and incubated for 1 h at room temperature with anti-rabbit or anti-mouse Alexa Fluor 488 or 594 (1:500, Molecular Probes). Slides were mounted with ProLong Gold antifade mounting reagent (Molecular probes) and cell staining was visualized with a fluorescence microscope using Zeiss filter sets #46 and #64HE (excitation band pass, 598/25 nm; emission band pass, 647/70 nm).

### LysoTracker-Red staining

MDA-MB 435S cells were loaded with 50 nM LysoTracker-Red for 20 min in the dark. After being washed with PBS, the cells were visualized by the fluorescence microscopy using Zeiss filter sets #64HE.

### Autophagy flux assay using mRFP/GFP LC3

MDA-MB 435S cells were seeded in a 12 well plate (1×10^5^ per well) on a cover slide and grown overnight. Cells were transfected with tandem mRFP/GFP-tagged LC3 using Lipofectamine PLUS reagent (Invitrogen) according to the manufacturer's recommendations. After incubation for 24 h, transfected cells were treated with or without bafilomycin A1, Torin1, celastrol for 8 h. Cells were visualized with a fluorescence microscope using Zeiss filter sets #46 and #64HE.

### Small interfering RNAs

MDA-MB 435S cells were transfected with ON-TARGETplus^TM^ SMARTpool siRNAs (Dharmacon), consisting of four pooled siRNA sequences rationally designed to minimized off-target effects using Lipofectamine 2000 (Invitrogen) according to the manufacturer's recommendations. The specific siRNA used in this study was MCU siRNA (siMCU, L-015519-02) and Negative Universal Control^TM^ (Invitrogen) was used as the control.

### Measurement of cytosolic and mitochondrial Ca^2+^ levels

To measure [Ca^2+^]_c_, treated cells were incubated with 2.5 μM Fluo-3-AM at 37°C for 20 min, washed with HBSS (without Ca^2+^ or Mg^2+^), and analyzed immediately by flow cytometry. To measure [Ca^2+^]_m_, treated cells were incubated with 2.5 μM Rhod-2-AM at 4°C for 30 min, washed with HBSS (without Ca^2+^or Mg^2+^), further incubated with HBSS at 37°C for 20 min, and then analyzed by flow cytometry and fluorescence microscopy. To confirm the mitochondrial localization of the Rhod-2 probe, cells were loaded with 2.5 μM Rhod-2-AM in HBSS (without Ca^2+^ or Mg^2+^) for 30 min at 4°C. The cells were then washed with HBSS and visualized by the fluorescence microscopy using Zeiss filter sets #46 and #20.

### Transmission electron microscopy

Cells were prefixed in Karnovsky's solution (1% paraformaldehyde, 2% glutaraldehyde, 2 mM calcium chloride, 0.1 M cacodylate buffer, pH 7.4) for 2 h and washed with cacodylate buffer. Post-fixing was carried out in 1% osmium tetroxide and 1.5% potassium ferrocyanide for 1 h. After dehydration with 50-100% alcohol, the cells were embedded in Poly/Bed 812 resin (Pelco), polymerized, and observed under electron microscope (EM 902A, Zeiss).

### Statistical analysis

All data were presented as mean ± S.D. (standard deviation) from at least three separate experiments. Student's *t* test was applied to evaluate the differences between treated and control groups with cell viability. Data from multiple groups were analyzed by one-way ANOVA, followed by Bonferroni multiple comparison test. For all the tests, the level of significance was values of *P* < 0.05.

### SUPPLEMENTAL MATERIAL AND FIGURES


