# Supplementary material for: Release of Ca2+ from the endoplasmic reticulum and its subsequent influx into mitochondria trigger celastrol-induced paraptosis in cancer cells
Source: Oncotarget. 2014 Jul 25;5(16):6816–31. doi: 10.18632/oncotarget.2256 (PMC4196165; doi:10.18632/oncotarget.2256)
Supplement: Supplementary file 1 [file oncotarget-05-6816-s001.pdf]

Release of Ca^{2+} from the endoplasmic reticulum and its subsequent influx into mitochondria trigger celastrol-induced paraptosis in cancer cells

Supplementary Material

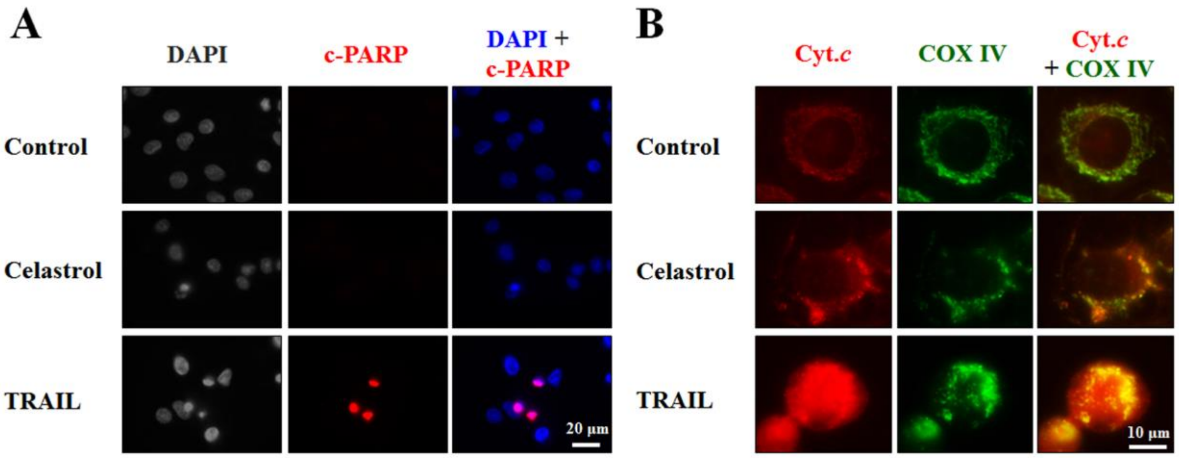

Supplementary Figure 1: Chromatin condensation, DNA fragmentation, PARP cleavage, and the release of mitochondrial cytochrome *c* are induced by TRAIL, but not by celastrol. (**A**) Immunocytochemistry using the antibody specific for the cleaved PARP (c-PARP) and DAPI staining were performed in MDA-MB 435S cells treated with 2 μM celastrol or 0.2 μg/ml TRAIL for 12 h, as described in Materials and Methods. Representative fluorescence microscopic images of cells are shown. (**B**) Immunocytochemistry of cytochrome *c* and the subunit I of cytochrome *c* oxidase (COX IV) was performed in MDA-MB 435S cells treated with 2 μM celastrol or 0.2 μg/ml TRAIL for 12 h. Representative fluorescence microscopic images of cells are shown.

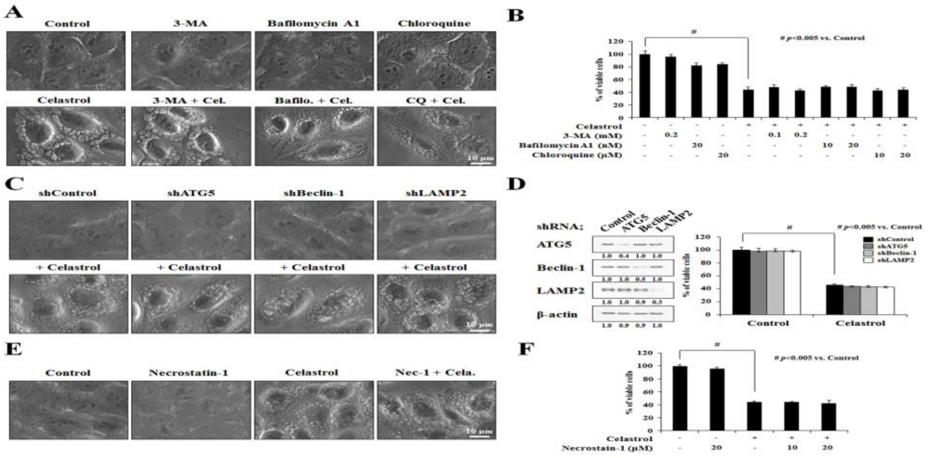

Supplementary Figure 2: Neither autophagy nor necroptosis is critically involved in celastrol-induced vacuolation and subsequent cell death. (**A**) MDA-MB 435S cells were pretreated with 100 μM 3-MA, 10 nM bafilomycin A1, or 10 μM chloroquine for 30 min and further treated with 2 μM celastrol for 16 h. Cells were observed under a phase contrast microscope. (**B**) MDA-MB 435S cells were left untreated or pretreated with the respective autophagy inhibitors (3-MA, bafilomycin A1, chloroquine) at the indicated concentrations for 30 min and further treated with 2 μM celastrol for 24 h. Cellular viability was assessed using calcein-AM and EthD-1. (**C**) MDA-MB 435S cells were treated with the lentivirus encoding the control non-targeting RNA, ATG5, Beclin-1 or LAMP2 shRNA and further treated with or without 2 μM celastrol for 8 h. Cell were observed under a phase contrast microscope. (**D**) MDA-MB 435S cells were treated with the lentivirus encoding the control non-targeting RNA, ATG5, Beclin-1 or LAMP2 shRNA and further treated with or without 2 μM celastrol for 24 h. Knockdown of these gene products was confirmed by Western blots (*left panel*). Cellular viability was assessed using calcein-AM and EthD-1 (right *panel*). (**E**) MDA-MB 435S cells were pretreated with 10 μM necrostatin-1 for 30 min and further treated with 2 μM celastrol for 8 h. Cells were observed under a phase contrast microscope. (**F**) MDA-MB 435S cells were pretreated with 10 μM necrostatin-1 for 30 min and further treated with 2 μM celastrol for 24 h. Cellular viability was assessed using calcein-AM and EthD-1.

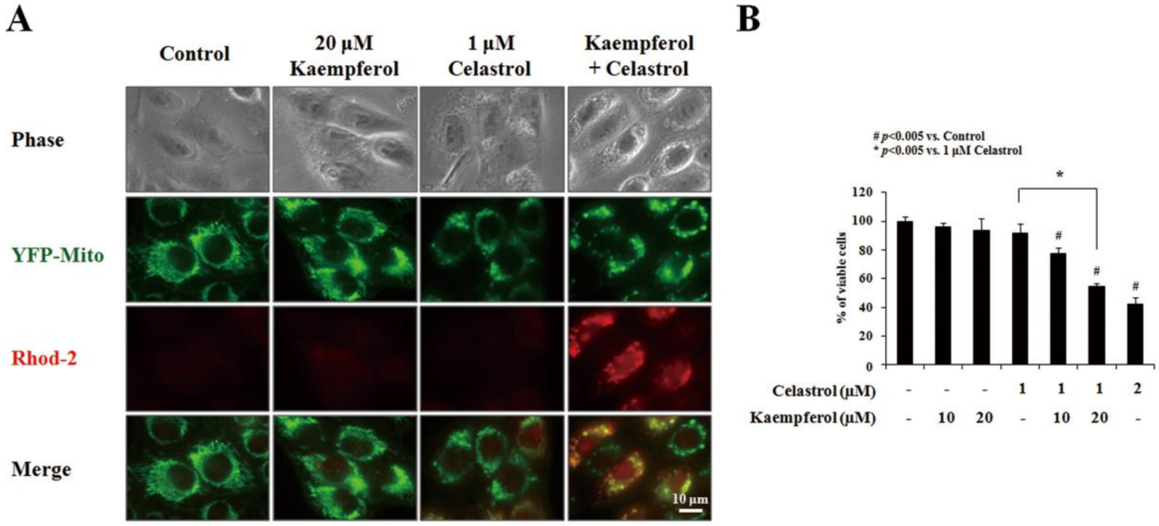

Supplementary Figure 3: Effect of kaempferol on celastrol-induced increase in $[Ca^{2+}]_m$ and cell death. (**A**) MDA-MB 435S cells were pretreated with or without 20 μM kaempferol, further treated with 1 μM celastrol for 2 h, stained with 2.5 μM Rhod-2 and and processed for the phase contrast and fluorescence microscopy. (**B**) MDA-MB 435S cells were pretreated with kaempferol at the indicated concentrations for 30 min and further treated with 1 μM celastrol for 24 h. Cellular viability was assessed using calcein-AM and EthD-1.

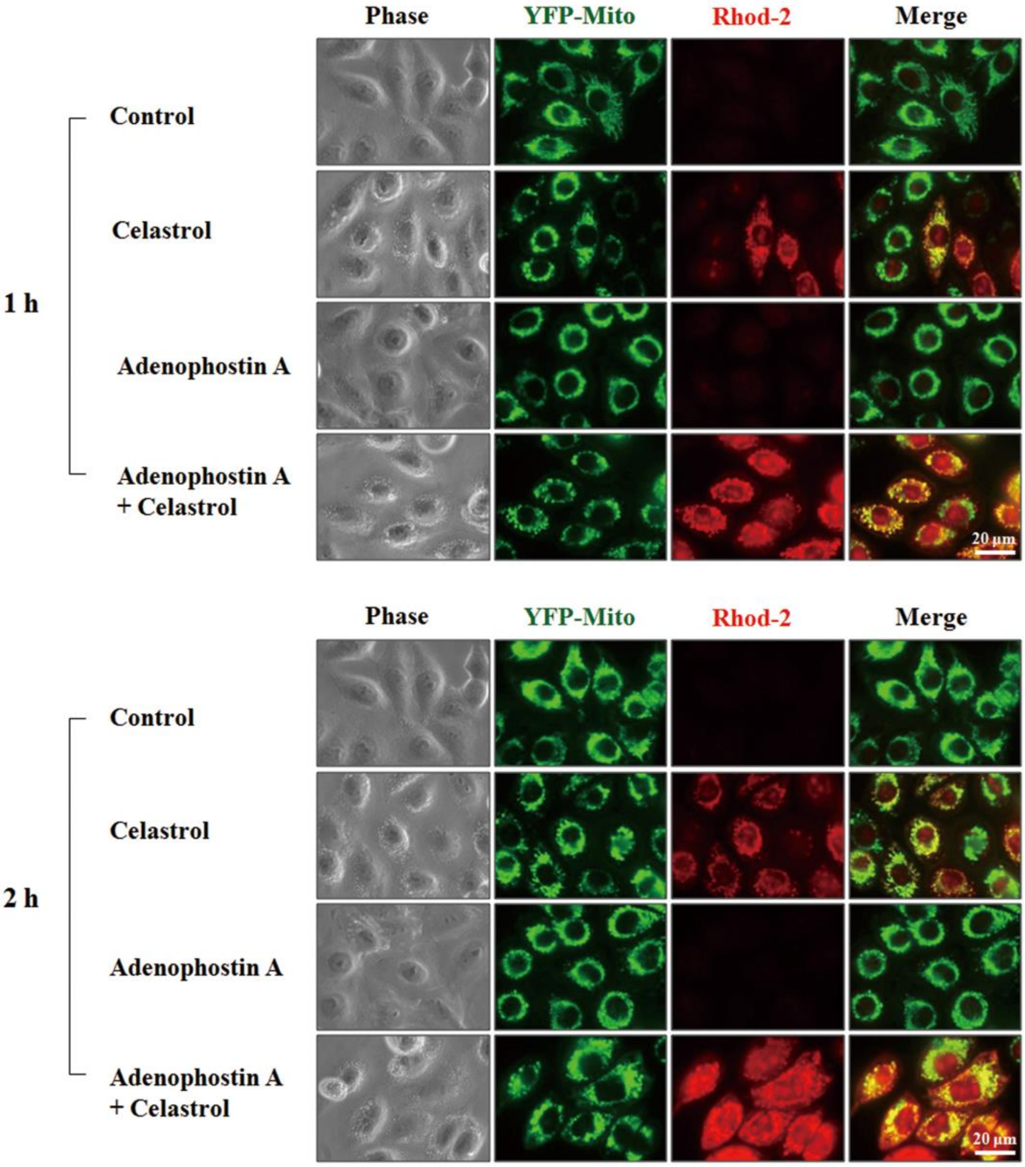

Supplementary Figure 4: Co-treatment with adenophostin A potentiated celastrol-induced mitochondrial Ca^{2+} accumulation. YFP-Mito cells were pretreated with or without 10 μM adenophostin A, further treated with 2 μM celastrol for 1 or 2 h, stained with 2.5 μM Rhod-2, and then observed under the phase contrast and fluorescence microscope.
